# *Legionella pneumophila* Infection Rewires the *Acanthamoeba castellanii* Transcriptome, Highlighting a Class of Sirtuin Genes

**DOI:** 10.3389/fcimb.2020.00428

**Published:** 2020-08-20

**Authors:** Pengfei Li, Dane Vassiliadis, Sze Ying Ong, Vicki Bennett-Wood, Chihiro Sugimoto, Junya Yamagishi, Elizabeth L. Hartland, Shivani Pasricha

**Affiliations:** ^1^Centre for Innate Immunity and Infectious Diseases, Hudson Institute of Medical Research, Clayton, VIC, Australia; ^2^Department of Microbiology and Immunology, University of Melbourne at the Peter Doherty Institute for Infection and Immunity, Melbourne, VIC, Australia; ^3^Cancer Research Division, Peter MacCallum Cancer Centre, Melbourne, VIC, Australia; ^4^Global Station for Zoonosis Control, GI-CoRE, Hokkaido University, Sapporo, Japan; ^5^Research Center for Zoonosis Control, Hokkaido University, Sapporo, Japan; ^6^Department of Molecular and Translational Science, Monash University, Clayton, VIC, Australia

**Keywords:** host-pathogen interaction, *Legionella pneumophila*, *Acanthamoeba castellanii*, transcription, Legionnaires' disease, gene expression

## Abstract

*Legionella pneumophila* is an environmental bacterium that has evolved to survive predation by soil and water amoebae such as *Acanthamoeba castellanii*, and this has inadvertently led to the ability of *L. pneumophila* to survive and replicate in human cells. *L. pneumophila* causes Legionnaire's Disease, with human exposure occurring via the inhalation of water aerosols containing both amoebae and the bacteria. These aerosols originate from aquatic biofilms found in artifical water sources, such as air-conditioning cooling towers and humidifiers. In these man-made environments, *A. castellanii* supports *L. pneumophila* intracellular replication, thereby promoting persistence and dissemination of the bacteria and providing protection from external stress. Despite this close evolutionary relationship, very little is known about how *A. castellanii* responds to *L. pneumophila* infection. In this study, we examined the global transcriptional response of *A. castellanii* to *L. pneumophila* infection. We compared *A. castellanii* infected with wild type *L. pneumophila* to *A. castellanii* infected with an isogenic Δ*dotA* mutant strain, which is unable to replicate intracellularly. We showed that *A. castellanii* underwent clear morphological and transcriptional rewiring over the course of *L. pneumophila* infection. Through improved annotation of the *A. castellanii* genome, we determined that these transcriptional changes primarily involved biological processes utilizing small GTPases, including cellular transport, signaling, metabolism and replication. In addition, a number of sirtuin-encoding genes in *A. castellanii* were found to be conserved and upregulated during *L. pneumophila* infection. Silencing of sirtuin gene, *sir6f* (ACA1_153540) resulted in the inhibition of *A. castellanii* cell proliferation during infection and reduced *L. pneumophila* replication. Overall our findings identified several biological pathways in amoebae that may support *L. pneumophila* replication and *A. castellanii* proliferation in environmental conditions.

## Introduction

The bacterial pathogen, *Legionella pneumophila* is foremost an environmental pathogen that replicates to high numbers within freshwater amoeba, such as *Acanthamoeba castellanii* (Atlas, 1999; Neumeister et al., 2000). It is only through “accidental” exposure to humans that *L. pneumophila* has caused outbreaks of the acute pneumonia, Legionnaire's disease (Best and Abu Kwaik, 2018). Human illness occurs through the inhalation of infected aerosols that originate from artificial water sources contaminated with *A. castellanii* and *L. pneumophila*, such as stagnant pipes, cooling towers, spas and fountains (Berk et al., 1998; Atlas, 1999; Newton et al., 2010). Upon inhalation, *L. pneumophila* is engulfed by alveolar macrophages and it is through mechanisms analogous to those employed during the infection of *A. castellanii* that *L. pneumophila* has become equipped to survive and replicate in humans (Winiecka-Krusnell and Linder, 1999; Greub and Raoult, 2004; Molmeret et al., 2005; Salah et al., 2009; Jager et al., 2014; Best and Abu Kwaik, 2018).

Within both amoebae and macrophages, *L. pneumophila* evades destruction by replicating within a “Legionella-containing vacuole” (LCV). LCV biogenesis depends on the secretion of more than 330 virulence (effector) proteins via the Dot/Icm secretion type IV secretion system (Burstein et al., 2009; Ensminger and Isberg, 2009; Huang et al., 2011; Zhu et al., 2011). Many effectors share similarity with eukaryotic proteins and interfere with multiple host cell processes through molecular mimicry (Hubber and Roy, 2010; Barlocher et al., 2017; Qiu and Luo, 2017; Mondino et al., 2020). Most recently, *L. pneumophila* effectors have been shown to modulate mammalian cell biology via Rab-GTPases, ubiquitination machinery, phosphoinositide metabolism, sphingosine metabolism and host transcription machinery (Mondino et al., 2020). Many of the eukaryotic-like effectors are believed to have been acquired by inter-kingdom transfer of genetic material during co-evolution of *L. pneumophila* with environmental amoebae (Best and Abu Kwaik, 2018).

Current measures for preventing outbreaks of Legionnaire's disease involve the monitoring and sterilization of artificial water supplies using biocides (Wang et al., 2012). Unfortunately, amoebae such as *A. castellanii* frequently develop resistance to these biocides (Turner et al., 2000; Coulon et al., 2010). This has resulted in outbreaks of Legionnaire's disease in aquariums, hotels, hospitals and aged care facilities worldwide (Srikanth and Berk, 1993; Fields et al., 2002; Garrison et al., 2016).

*A. castellanii* is thought to maintain a bi-phasic life cycle. Under nutrient rich conditions *A. castellanii* grows as vegetative trophozoites and replicates through binary-fission, while under nutrient limited or stressful conditions it takes on a dormant cyst form. Characteristically, cysts are double-membraned, creating a strong barrier between the amoeba and external stresses (Chávez-Munguía et al., 2013). The *A. castellanii* (Neff) genome contains approximately 15,450 intron-rich genes, a number of which are believed to have arisen from extensive horizontal gene transfer between *A. castellanii* and bacteria, archaea, viruses and other eukaryotes (Clarke et al., 2013). Importantly, most of the intron-rich genes are both expressed and incorporated into *A. castellanii* transcriptional programs (Clarke et al., 2013). The polyploid organization of the *A. castellanii* genome has made genetic manipulation, including the creation of gene knockouts particularly challenging (Swart et al., 2018). Therefore, much of the amoebae-*Legionella* relationship has been inferred from work in the genetically tractable but distantly related amoeba, *Dictyostelium discoideum*, which is not a natural host of *L. pneumophila* (Farbrother et al., 2006; Taylor et al., 2009; Bretschneider et al., 2016; Kjellin et al., 2019).

With advances in molecular strategies such as RNA interference and high-throughput sequencing, researchers are beginning to dissect the intra-*A. castellanii* stage of the *L. pneumophila* life cycle (Lorenzo-Morales et al., 2005; Swart et al., 2018). Studies have shown that *A. castellanii* supports *Legionella* replication, protects the bacteria from external stress and passage through *A. castellanii* results in increased virulence potential (Cirillo et al., 1994; Barker et al., 1995). During *L. pneumophila* infection, *A. castellanii* cells become rounded, cell division and replication of the amoebae are restricted and ultimately *A. castellanii* is killed by replicating *L. pneumophila* (Mengue et al., 2016).

In this study we employed RNA-sequencing technologies and extensively annotated the *A. castellanii* (Neff) genome bioinformatically to understand how *A. castellanii* responds to *Legionella* infection. Experiments were conducted at 37°C to simulate the warm conditions found in many artificial water systems and to allow for the comparison of different host responses. We identified clear morphological and transcriptional changes in *A. castellanii* over the course of infection. Genes differentially expressed during infection fell into fundamental biological processes, such as small GTPase signaling, cytoskeleton dynamics, metabolism and cell proliferation. Members of the Sirtuin family of class III histone deacetylases were upregulated by *A. castellanii* throughout wild type *L. pneumophila* infection. *sir6f* expression was important for *A. castellanii* proliferation during infection and *L. pneumophila* replication and presented as a possible candidate for amoebae-targeted anti-microbial control.

## Materials and Methods

### Bacteria Strains and Growth Conditions

Bacterial strains used in this study are listed in Table S1. *L. pneumophila* strains were cultured in ACES [N-(2-acetamido)-2-aminoethanesulfonic acid] broth or on buffered charcoal yeast extract (BCYE) agar, supplemented with 6 μg/ml chloramphenicol when required and incubated at 37°C.

### *A. castellanii* Cell Culture

*A. castellanii Neff* strain (ATCC® 30010™) was cultured in Peptone Yeast Glucose medium (PYG) (2% peptone, 0.1% sodium citrate, 0.1% yeast extract) supplemented with 0.1 M glucose, 0.4 mM CaCl_2_, 2.5 mM KH_2_PO_4_, 4 mM MgSO4, 2.5 mM Na_2_HPO_4_, 0.05 mM Fe_4_O_21_P_6_ at room temperature (RT). When infected with *L. pneumophila, A. castellanii* was incubated in minimal medium (PYG media without glucose, yeast extract or tryptone) at 37°C.

### *L. pneumophila* Intracellular Replication Assay

2 × 10^5^ *A. castellanii* were seeded in technical and biological triplicate in PYG medium in 24-well plates (4 × 10^5^ cells per ml) and incubated for 24 h at RT. *A. castellanii* was then washed with minimal medium to remove non-adherent cells (Coil et al., 2008). *A. castellanii* was infected with stationary phase *L. pneumophila* (MOI 2) and incubated at 37°C with 5% CO_2_. After 2 h, the media was replaced with minimal medium containing gentamicin (100 μg/mL) and incubated for 1 h at 37°C. Media was then removed and replaced with fresh minimal medium. At desired time-points, bacteria were released from amoebae by vigorous pulse vortex. Serial dilutions of the inoculum and bacteria recovered from lysed amoebae were plated on BCYE agar and incubated at 37°C for 72 h. Bacterial colony forming units (CFU) were then enumerated.

### *A. castellanii* Proliferation Assay

*A. castellanii* proliferation was assessed according to previously published protocol (Mengue et al., 2016). Briefly, 1 × 10^4^ cells were seeded in technical and biological triplicate in 24 well plates and infected with *L. pneumophila* (MOI 20) in minimal medium. After 2 h infection, the medium was replaced with PYG containing 20 μg/ml gentamicin. After 16, 24, and 48 h cells were harvested and enumerated using a hemocytometer.

### Electron Microscopy

8 × 10^5^ *A. castellanii* were seeded into 6-well plates, infected with *L. pneumophila* (MOI 20) in minimal medium and incubated at 37°C. After 2 h the medium was replaced with PYG. 8, 16, and 24 h post infection cells were harvested, pelleted by centrifugation (10,000 g, 2 min), resuspended in fixation buffer (2.5% glutaraldehyde in 0.1 M sodium cacodylate) and incubated for 2 h at RT. Cells were then pelleted by centrifugation and washed twice with 0.1 M sodium cacodylate before post-fixation in 1% osmium tetroxide for 2 h at RT. Cell pellets were then washed in dH_2_O and left overnight at 4°C in 0.3% uranyl acetate. Samples were rinsed in dH_2_O before dehydration in a graded series of acetone and then infiltrated and embedded with EPON resin. Sections (70–80 nm thick) were cut, stained with uranyl acetate and lead citrate before viewing under a Phillips CM120 transmission electron microscope at 120 Kv.

### RNA Isolation

2 × 10^5^ *A. castellanii* were seeded in technical and biological triplicate in PYG medium in 24-well plates (4 × 10^5^ cells per ml) and incubated for 24 h at RT. *A. castellanii* was then washed with minimal medium to remove non-adherent cells followed by *L. pneumophila* infection with MOI 40 (Coil et al., 2008). After 1 h incubation at 37°C with 5% CO_2_, cells were washed with minimal medium and incubated in fresh PYG medium at 37°C with 5% CO_2._ For RNA-sequencing analysis RNA was isolated from *A. castellanii* using TRIsure™ (Bioline) according to manufacturer's instructions at 3, 8, 16, and 24 h post infection.

For qRT-PCR, RNA was isolated using Isolate II RNA Mini Kit (Bioline) according to manufacturer's instructions.

### Library Preparation, Sequencing, and Alignment

For RNA sequencing, cDNA library preparation and sequencing was carried out as described previously (Tanaka et al., 2013). Briefly, 1 μg RNA was used for poly(A) selection and cDNA libraries were prepared using the Illumina sample preparation kit. 100 base pair paired-end sequencing was performed on the HiSeq 2500 (Illumina) platform, with the use of TruSeq Stranded mRNA LT sample Prep Kit (IIIumina) according to manufacturer's instructions. Raw sequence reads were mapped to the *A. castellanii* (ATCC 30010) genome using Tophat2 v2.1.1 (Kim et al., 2013) and HTSeq was used to count reads (Anders et al., 2015).

### Differential Gene Expression Analysis and Clustering

Principal component analysis (PCA) and differential gene expression analysis was performed using the R software package, DeSeq2 (Love et al., 2014). Volcano plots were created using Excel. Plots were used to determine 5% FDR cut-offs. Genes that showed significant log_2_ fold-change of < −2 or > 2 for at least 1 time point were selected for subsequent clustering analysis. k-means clustering analysis with Pearson correlation and heatmap visualization was performed with Multi experiment Viewer MeV, Version 4.8 (Saeed et al., 2006).

### Genome Annotation and Gene Ontology

The *A. castellanii* genome was annotated using BlastXfast and InterPro (www.ebi.ac.uk/interpro/). Blast results were merged and mapped using Blast2GO (Annex). Gene ontology (GO) terms were allocated to each putative protein-encoding gene in *A. castellanii* using BLAST X, Interproscan, GO-EnzymeCode and KEGG search results and mapped with GO-slim in Blast2GO (Conesa et al., 2005; Conesa and Gotz, 2008; Gotz et al., 2008, 2011). These GO assignments were then used to examine gene relationships within each cluster using GoTermFinder (http://go.princeton.edu/cgi-bin/GOTermFinder) and the statistical significance determined by *p*-value (*p* < 0.05) using a hypergeometric distribution that takes into account bias in the sample population (Boyle et al., 2004).

### cDNA Synthesis and qRT-PCR

For qRT-PCR experiments cDNA was synthesized using iScript^tm^ cDNA synthesis kit (Bio-Rad) according to the manufacturer's instruction. The product was used as the template for qRT-PCR without dilution. qRT-PCR was performed on QuantStudio^tm^ Version 6.0 q-RT-PCR system. Each 10 μL qRT-PCR reaction consisted of 2 μL cDNA template, 300 nM of each primer and 5 μL Sso-Advanced Universal SYBR Green Supermix (BioRad). The mixture was loaded into MicroAmp® Optical 384-Well Reaction Plate (Life technologies # 4309849) and cycled as follows: 95°C for 10 min, followed by 40 cycles of [95°C for 15 s, 60°C for 60 s]. Data were analyzed by QuantStudio^tm^ real-time PCR program using 18s rRNA as the endogenous control and uninfected samples as the reference sample. Results were expressed as the mean of at least three independent biological repeats.

### Transient Transfection and Gene Silencing

Small interfering RNA (siRNA) targeting *sir6f* (ACA1_154540) mRNA was synthesized by Bioneer. The duplex siRNA with sense (5'- GACAUCAAGGAGGUGGAGU) and antisense (5′-ACUCCACCUCCUUGAUGUC) was hydrated in sterile siRNA buffer to a final concentration of 10 μM. Non-targeting scramble siRNA (Bioneer) optimized for human and mouse cell lines was used as a knockdown control. SuperFect (Qiagen) was used for siRNA transfection in *A. castellanii*. Generally, 25 μL PYG was mixed with 5 μL SuperFect and 2.5 μL siRNA, and then incubated for 10 min at RT. Meanwhile, 8 × 10^4^ *A. castellanii* were seeded in PYG medium in 24-well plates (1.6 × 10^5^ cells per ml). The mixture of siRNA and SuperFect was added to the *A. castellanii* culture and incubated at RT for 48 h.

After siRNA transfection, *L. pneumophila* infection was carried out as described above but with modification. Briefly, the siRNA transfected *A. castellanii* were infected with *L. pneumophila* with a MOI of 40. After 1 h incubation at 37°C, cells were treated with gentamycin (100 μg/mL) for another hour. Cells were then harvested and washed with minimal medium and incubated in PYG. The knock-down of *sir6f* was validated at 24 h post infection by qRT-PCR. *A. castellanii* proliferation was measured after siRNA transfection and *L. pneumophila* infection using haemocytometer. *A. castellanii* viability was also measured by trypan blue (Invitrogen™). 10 μl cell suspension was mixed with 10 μl 0.4% trypan blue. Dead cells were enumerated using a haemocytometer (Sigma) within 3 min. 100 cells were measured for each sample. *A. castellanii* treated with 4% PFA was used as a positive control for the viability assay.

### Bioinformatic Analysis of *A. castellanii* Protein Homologs

Protein homologs were searched by BlastP (NCBI) against select protein sequence databases in Genbank (http://blast.ncbi.nlm.nih.gov/Blast.cgi). All recruited significant homologs were aligned using ClustalW and the resulting alignment was used to perform molecular phylogenetic analysis by maximum likelihood method in MEGA7 (Kumar et al., 2016). The evolutionary history was inferred by using the Maximum Likelihood method based on the Poisson correction model (Zuckerland and Pauling, 1965). The tree with the highest log likelihood (−40715.42) is shown. Initial tree(s) for the heuristic search were obtained automatically by applying Neighbor-Join and BioNJ algorithms to a matrix of pairwise distances estimated using a JTT model, and then selecting the topology with superior log likelihood value.

## Results

### Gross Morphological Changes in *A. castellanii* Upon Infection With *L. pneumophila*

**Figure 1:**
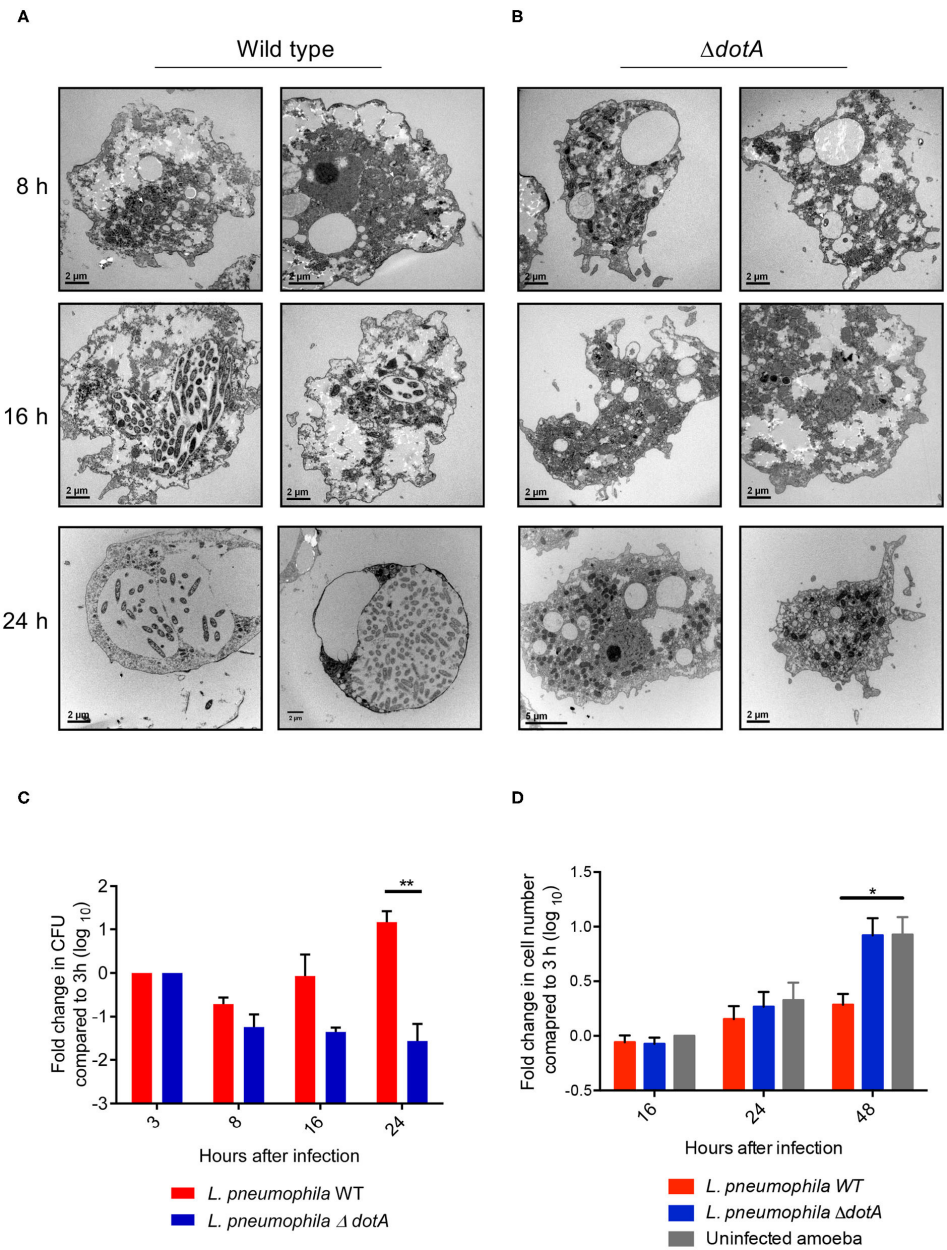
Changes in *A. castellanii* morphology and proliferation during *L. pneumophila* infection. **(A)** Electron micrographs of *A. castellanii* infected with wild type (130b) or **(B)** Δ*dotA L. pneumophila* (MOI 10) taken at 8, 16, and 24 h post infection. Two representative images of each condition are depicted. **(C)** *A. castellanii* was infected with wild type or the Δ*dotA* mutant strain of *L. pneumophila* 130b (MOI 10) and incubated at 37°C. Amoeba were lysed and the *L. pneumophila* plated 3, 8, 16, and 24 h post infection. The *L. pneumophila* colony forming units (CFUs) are presented as a fold change in CFUs relative to 3 h post infection. Error bars represent the standard error of the mean, where n = 5 and **p < 0.01. **(D)** *A. castellanii* was infected with wild type or the Δ*dotA* mutant strain of *L. pneumophila* 130b (MOI 10), or uninfected, and incubated at 37°C. The number of *A. castellanii* cells per well were quantified 0, 16, 24, and 48 h post infection. Data presented as a fold changed in cell numbers relative to 0 h. Error bars represent the standard error of the mean, where *n* = 3 and *p < 0.05, **p < 0.01.

Using electron microscopy (EM) we observed that, similar to recent studies performed using the *Philadelphia* strain of *L. pneumophila* (Mengue et al., 2016), infection of *A. castellanii* trophozoites with wild type *L. pneumophila* (130b) resulted in clear morphological changes of *A. castellanii* from 16 h post infection (Figure 1A). *A. castellanii* appeared rounded compared to uninfected trophozoites, and lacked thick cell membrane structures, suggesting the amoebae had not transitioned to mature cysts. Importantly, *A. castellanii* infected with the mutant *L. pneumophila* strain, Δ*dotA*, which is unable to translocate Dot/Icm effector proteins and establish the LCV, appeared similar to uninfected *A. castellanii* (Figure 1B), whereby *A. castellanii* remained adherent and maintained trophozoite-like morphology. Consistent with Mengue et al. (2016), we saw that wild type *L. pneumophila*, unlike Δ*dotA*, continued to replicate in *A. castellanii* cells for up to 48 h, suggesting that the changes in *A. castellanii* morphology observed from 16 h onwards did not impede *L. pneumophila* replication (Figure 1C). In contrast, proliferation of *A. castellanii* was reduced after 48 h of infection with wild type *L. pneumophila* compared to infection with Δ*dotA* or uninfected cells (Figure 1D).

### Temporal Transcriptomic Changes in *A. castellanii* Following *L. pneumophila* Infection

To determine how *A. castellanii* responds to *L. pneumophila* infection at the transcriptional level we performed RNA-sequencing analysis on *A. castellanii* trophozoites left uninfected or infected with wild type *L. pneumophila* or the isogenic Δ*dotA* mutant for 3 h, 8 h (early infection), 16 h (mid infection) or 24 h (late infection). *A. castellanii* was infected with *L. pneumophila* strains at MOI 40, RNA was extracted using TRIsure, followed by poly(A) selection and cDNA library construction (Figure S1). Samples were sequenced on the HiSeq 2500 (Illumina) platform and resulting reads were aligned to the *A. castellanii* (Neff) genome. Three biological repeats for each condition were included in the study, however one 16 h wild type *L. pneumophila* infected *A. castellanii* repeat was removed from subsequent analysis due to contamination.

**Figure 2:**
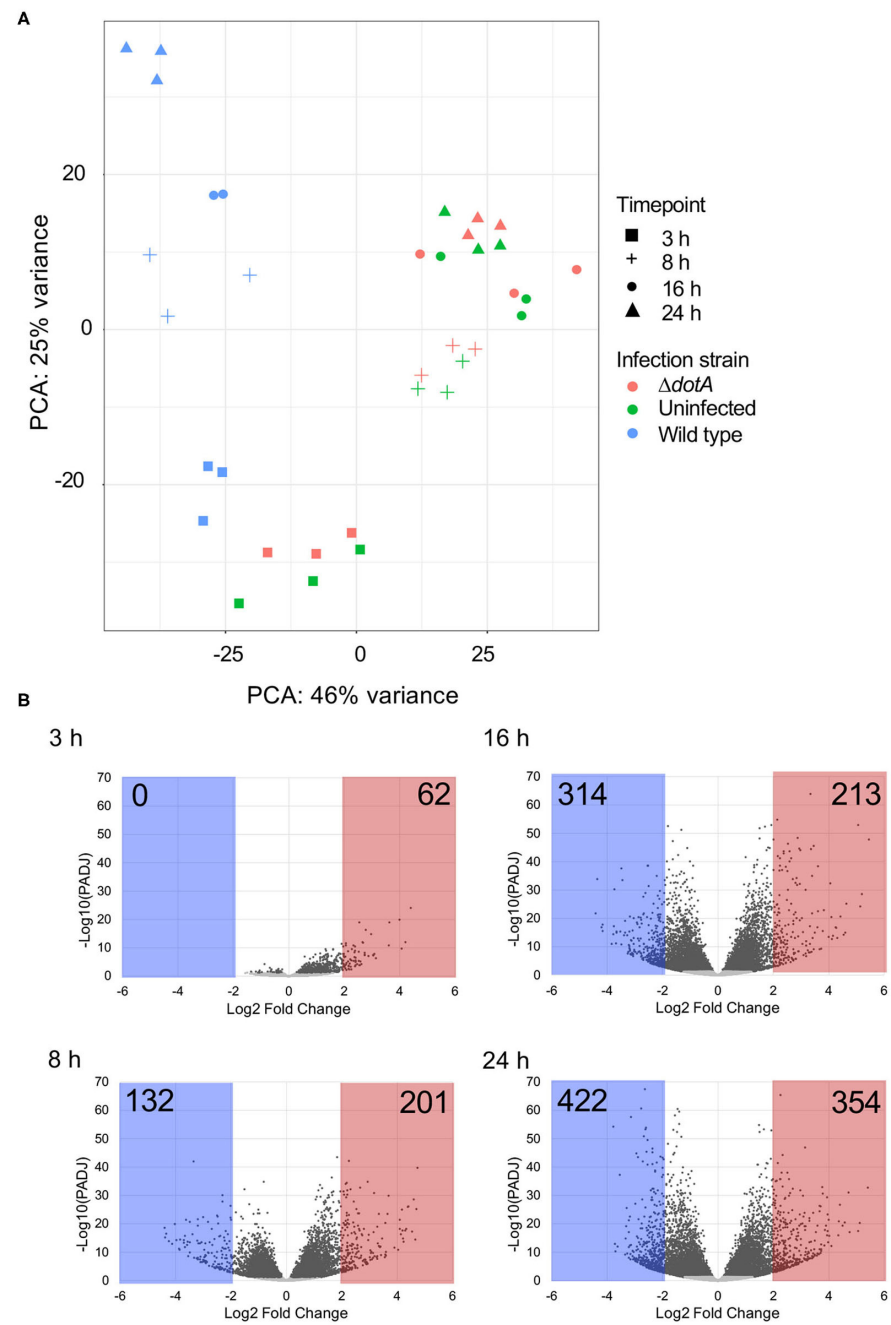
Differential gene expression of *A. castellanii* upon *L. pneumophila* infection. **(A)** Principal components analysis (PCA) showing the relatedness of sample transcriptomes across 2 principal component axes. Different strains are distinguished by color and time points by shape. **(B)** Global changes in *A. castellanii* gene expression after wild type *Legionella* infection relative to infection with Δ*dotA L. pneumophila* (WT vs. Δ*dotA*) at 3, 8, 16, and 24 h post infection. Volcano plots illustrate the distribution of change [log2(fold-change)] detected against significance, *p-*value adjusted for multiple testing using Benjamini–Hochberg to estimate the false discovery rate (PADJ). Points shaded gray represent genes with a PADJ > 0.05. These genes were omitted from subsequent analysis. Genes with a log2(fold-change) difference of > 2 are shaded red and < −2 are shaded blue.

Principal components analysis (PCA) revealed clear separation of *A. castellanii* samples infected with wild type *L. pneumophila* compared to uninfected cells or those infected with Δ*dotA* across the 24 h period (Figure 2A). Comparison of the expression profiles of wild type *L. pneumophila* infected vs. Δ*dotA* infected *A. castellanii* revealed that after 3 h of infection, 4.1% expressed genes showed statistically significant differential expression upon wild type infection compared to Δ*dotA* (5% false discovery rate [FDR] cut-off and adjusted *p* < 0.05) (Figure 2B, Tables S2, S3). After 8 h, this increased to 45.2%, after 16 h, 54%, and by 24 h, 55.8% of expressed genes showed statistically significant differential expression between wild type and Δ*dotA* infection (Figure 2B). *A. castellanii* genes that showed a fold-change in expression of >4 or <1/4 for wild type vs. Δ*dotA* infection were selected for further analysis (62 upregulated genes at 3 h, 132 downregulated genes and 201 upregulated at 8 h, 314 downregulated and 213 upregulated genes at 16 h, 422 downregulated and 354 upregulated genes at 24 h) (Figure 2B).

**Figure 3:**
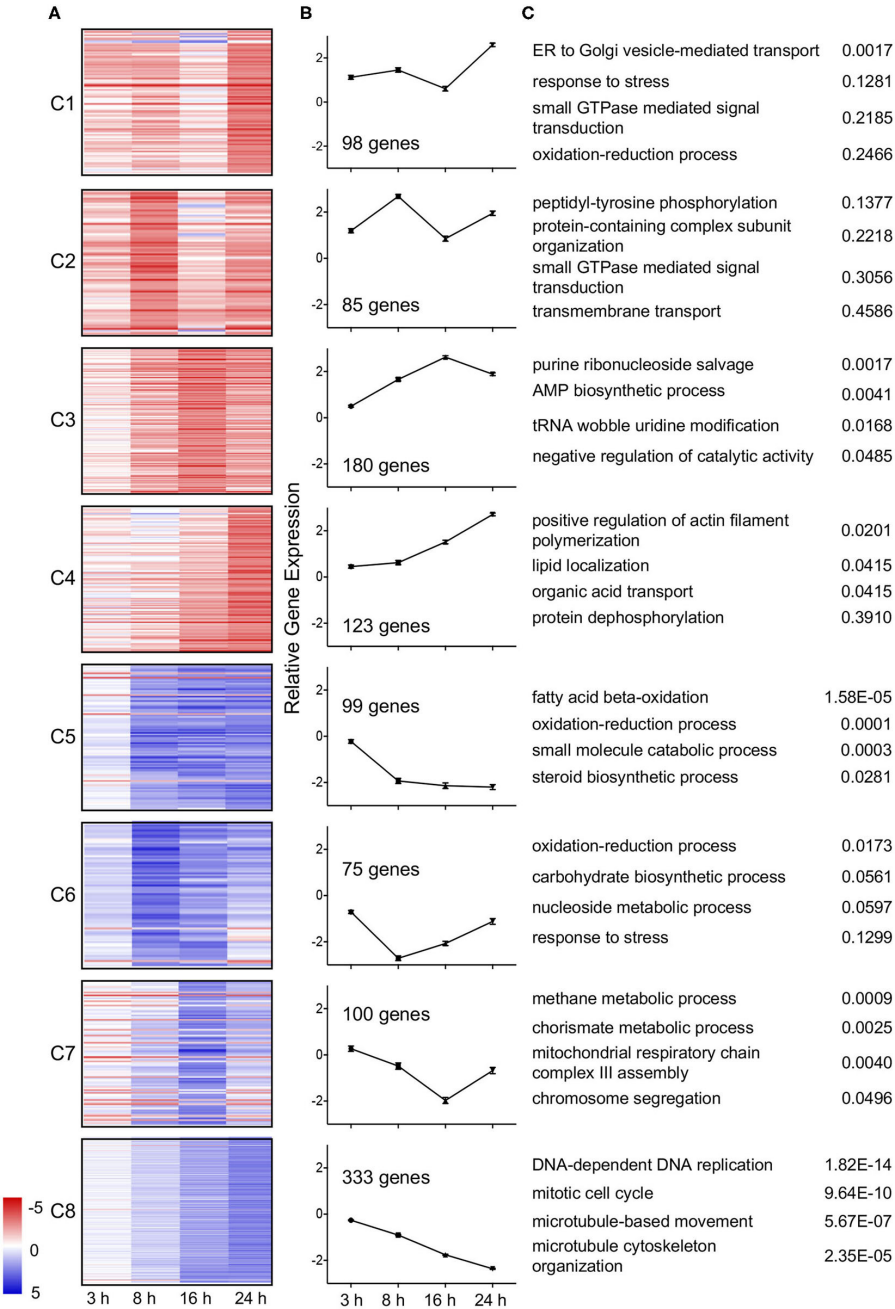
Temporal clustering of differential expressed genes of wild type *L. pneumophila* infected *A. castellanii* compared to Δ*dotA* infected *A. castellanii*. Eight temporal gene expression clusters based on k-means clustering of genes that were significantly differentially expressed between wild type *L. pneumophila* vs. Δ*dotA* mutant infected *A. castellanii* for at least one timepoint (fold-change of >4 or <1/4, PADJ < 0.05). **(A)** Heatmaps showing the log2 fold change in expression [blue (−5)—red (+5)] of the individual genes in cluster C1-C8. **(B)** Mean differential expression over time **(C)** GO enriched biological processes for each cluster and uncorrected *p*-value.

Temporal trends in gene expression were further examined by k-means clustering using MeV (Saeed et al., 2006). Expression data for genes differentially expressed in wild type *L. pneumophila* infected *A. castellanii* vs. Δ*dotA* infected *A. castellanii* were clustered into 8 temporal expression profiles using Pearson correlation (Figure 3). Genes included in the clusters were differentially expressed (fold-change of >4 or <1/4) during at least one timepoint. Four clusters were formed from upregulated genes. Genes in clusters C1 (8.97% of genes) and C4 (11.25%) exhibited maximal upregulation late in the infection cycle, between 16 h and 24 h post infection. In contrast, cluster C2 (7.78%) genes showed peak induction at 8 h followed by a reduction by 24 h. Cluster C3 (16.5%) showed gradual upregulation up to 16 h post infection. Finally, cluster C4 genes showed increasing expression over time.

Of the four downregulated gene clusters, cluster C5 (9.06%) genes were downregulated from 8 h at a consistent level. Cluster C6 (6.86%) genes showed a reduction in expression early in infection (3 h and 8 h) and were upregulated as the infection progressed (16 and 24 h). Cluster C7 (9.15%) genes were downregulated from 3 to 16 h but were then induced at 24 h (C7). Finally, cluster C8 (30.47%) genes showed a gradual decrease in expression over time (Figure 3). Identified trends in gene expression may reflect changes in growth and infection pressures faced by *A. castellanii* over the duration of the *L. pneumophila* infection.

### Upregulation of Vesicle Transport and Small GTPase-Mediated Signal Transduction

To understand the biological impact of *L. pneumophila* infection on *A. castellanii*, differentially regulated genes were analyzed using Gene ontology (GO) (Princeton). Despite evidence of lateral gene transfer from a number of kingdoms, previous GO term annotation of the *A. castellanii* genome has been based on GO term assignments of only closely related organisms (Clarke et al., 2013). In this study we have accounted for a wide range of possible GO term allocations by using current databases, BlastXfast and InterPro. Blast results were mapped and GO terms assigned using Blast2GOPro (Version 5.1). This increased the GO term allocation by approximately 2000 terms (Table S4). GO enrichment analysis was performed on temporal clusters (C1–C8) and showed that distinct biological processes were upregulated or downregulated at different stages of infection (Figure 3).

A subset of *A. castellanii* genes predicted to be involved in ER to Golgi vesicle transport showed a dramatic increase in expression late in infection (24 h) (C1, Figure 3). Another subset of genes involved in small GTPase signaling was also upregulated, specifically at 8 and 24 h post infection (C2, Figure 3). Other subsets upregulated throughout the infection period (C4) included genes for actin filament polymerization and lipid localization (Figure 3).

**Figure 4:**
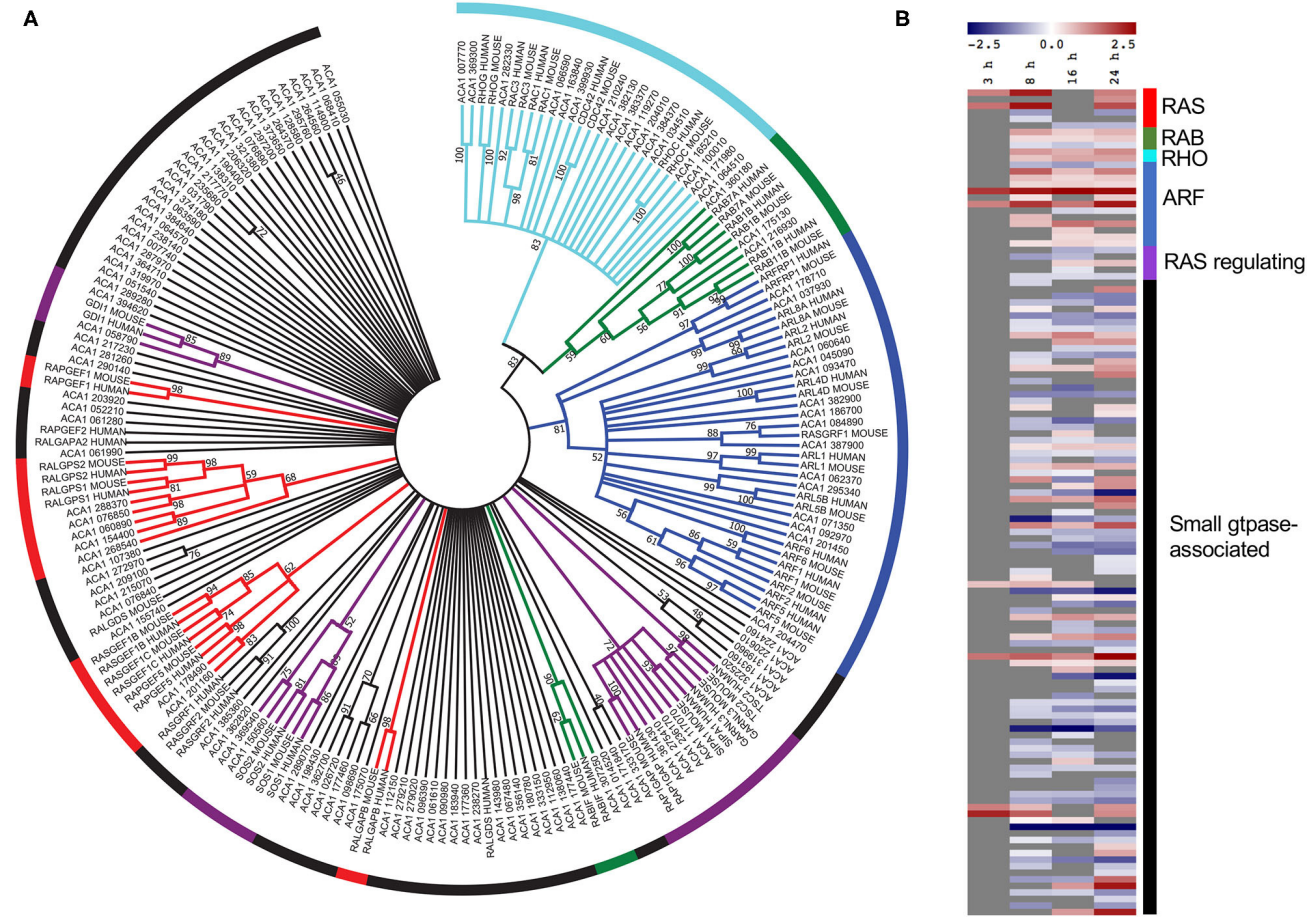
Conservation and differential gene expression of small GTPase-encoding genes in *L. pneumophila* infected *A. castellanii*. **(A)** *A. castellanii* proteins (ACA1) allocated the GO-term “small GTPase-mediated signal transduction” were selected for BlastP analysis against *Homo sapiens* (human) and *Mus musculus* (mouse), and the top protein hit (*p* < 0.01) selected. Multiple protein sequence alignment was conducted using ClustalW and the relatedness tree was created using the Maximum Likelihood method in MEGA7 (Kumar et al., 2016). Numbers next to the branching points indicate the relative support from 500 bootstrap replicates (only scores above 40 are shown). **(B)** Heatmap showing the log2(fold change) in gene expression of wild type *L. pneumophila* vs. Δ*dotA* mutant infected *A. castellanii* genes with GO-term “small-GTPase.” Levels of expression depicted from Blue (−5)—Red (+5), and gray represents insignificant differential expression (PADJ > 0.05).

Given that small GTPase associated genes play a critical role during *L. pneumophila* infection of mammalian hosts we were interested in examining this family of genes in *A. castellanii* further (Neunuebel and Machner, 2012; Hilbi et al., 2014). All *A. castellanii* proteins annotated with the processes-related GO term “small GTPase-mediated signal transduction” (GO:0007264) (125 proteins) were aligned with human and mouse small GTPases using ClustalW, and a phylogenetic tree was constructed by maximum likelihood (500 bootstrap replicates) (Figure 4A). Results suggested that while some *A. castellanii* protein homologs branched with specific small GTPase protein families- Ras, Rho, Rab, and Arf, and small GTPase regulators (49 proteins), there were a number of *A. castellanii* proteins that appeared to be “small GTPase-like,” homologs that did not branch with a specific family (76 proteins) (Figure 4A). Consistent with the GO enrichment analysis a number of small-GTPase associated genes were upregulated during early (8 h) and/or late infection (24 h) (Figure 4B). Interestingly, proteins that were clear homologs to Rho, Rab and Arf GTPases showed upregulation of gene expression throughout infection, whereas Ras GTPase homologs were upregulated at 8 h and 24 h post infection, while Ras regulators and small GTPase-like genes showed no convincing trend in gene expression (Figure 4B).

### Downregulation of Energy Metabolism and Cell Cycle Throughout Infection

During early infection (between 3 and 8 h) approximately 16% of differentially regulated *A. castellanii* genes underwent significant downregulation. Of this subset, approximately 1/3 remain downregulated at a constant rate (C5) or begin to increase in expression from 8 h (C6), or 16 h (C7). All three of these downregulated subgroups showed significant GO enrichment in metabolic processes, including fatty acid beta-oxidation, carbohydrate biosynthesis, as well as mitochondrial respiratory chain complex III assembly (Figure 3). These findings are consistent with the early (6 h) transcriptional profile of *D. discoideum* infected with *L. pneumophila* (*Philadelphia* I JR32), where a reduction in acetyl-CoA metabolism and mitochondrial electron transfer chain associated genes was observed (Farbrother et al., 2006; Kjellin et al., 2019).

Other *A. castellanii* genes that were increasingly downregulated over the course of the wild type *L. pneumophila* infection (C8) showed strong GO enrichment for DNA replication processes, including DNA-dependent DNA replication, mitotic cell cycle and associated microtubule-based movement. This finding was consistent with the reduction in *A. castellanii* proliferation observed at 24 h to 48 h following wild type *L. pneumophila* infection (Figure 1C).

### Role of Sirtuin-Encoding Genes During *L. pneumophila* Replication in *A. castellanii*

**Figure 5:**
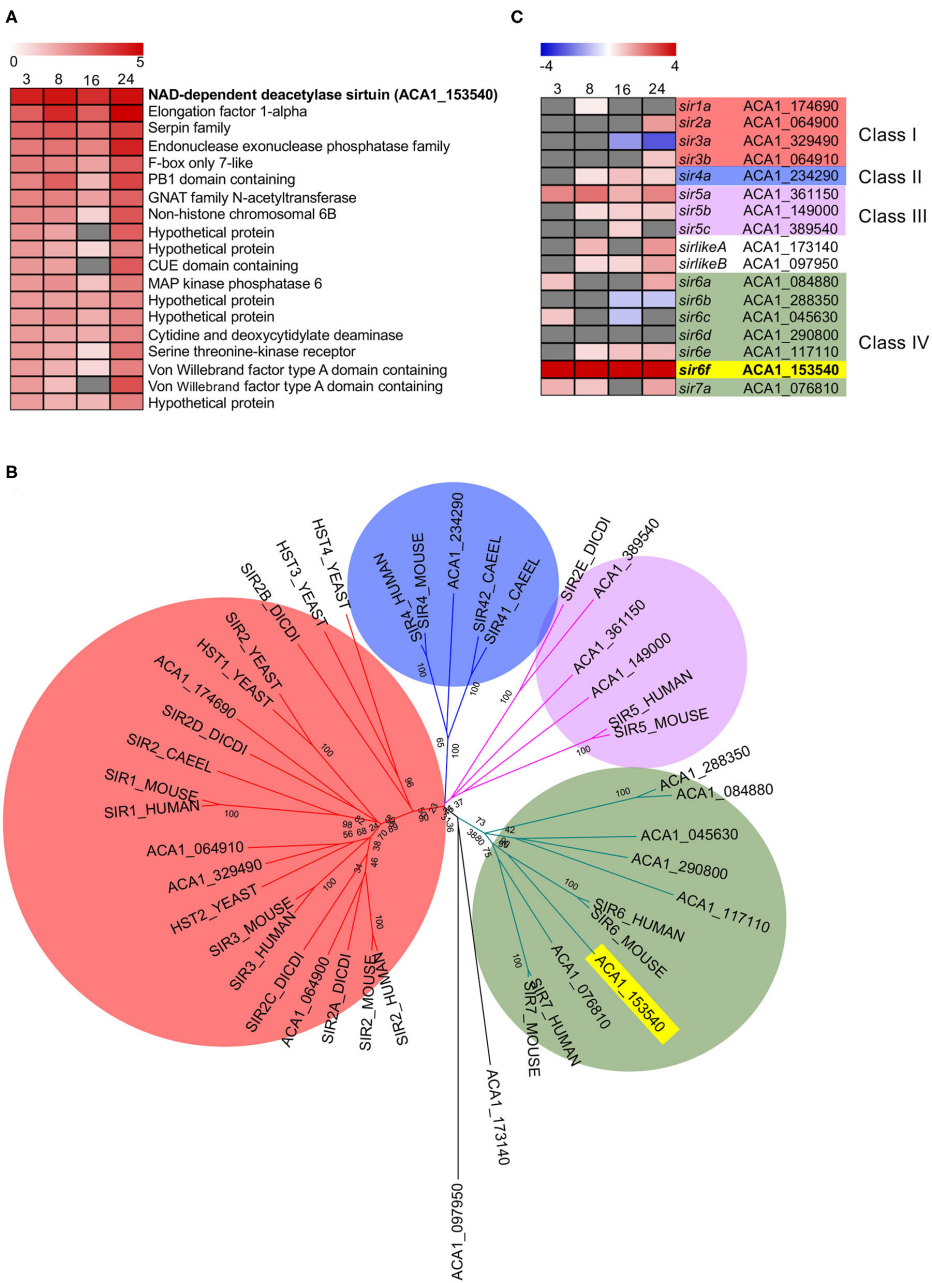
Conservation and differential gene expression of sirtuin-encoding genes in *L. pneumophila* infected *A. castellanii*. **(A)** Heatmap of Top 20 differentially upregulated genes comparing wild type *L. pneumophila* vs. Δ*dotA* mutant infected *A. castellanii* from Cluster 1 (Figure 3). Gray represents insignificant differential expression (PADJ > 0.05) **(B)** BlastP of *A. castellanii* proteome against selected eukaryotes was performed and the top protein hit (*p* < 0.01) selected. Multiple protein sequence alignment was conducted using ClustalW and the relatedness tree was created using the Maximum Likelihood method in MEGA7 (Kumar et al., 2016). Numbers next to the branching points indicate the relative support from 1,000 bootstrap replicates. Eukaryotes included are *A. castellanii* (ACA1), *Homo sapiens* (human), *Mus musculus* (mouse), *Caenorhabditis elegans* (Caeel), *Sacchromyces cerevisiae* (yeast), *D. discoideum* (Dicdi). **(C)** Heatmap showing the log2(fold change) in gene expression of wild type *L. pneumophila* vs. Δ*dotA* mutant infected *A. castellanii* putative sirtuin encoding genes. Levels of expression depicted from Blue (−5)—Red (+5), and gray represents insignificant differential expression (PADJ > 0.05). Yellow and bold text highlights the overall gene of interest, *sir6f* (ACA1_153540).

Inspection of the top 20 most upregulated *A. castellani* genes from C1 (Figure 3) highlighted a number of genes that did not fall into GO-enriched biological processes but may play important roles during infection (Figure 5A). At all the time points inspected, ACA1_153540 was upregulated in *A. castellanii* infected with wild type *L. pneumophila* compared to Δ*dotA* infected amoebae (Figure 5A). This was confirmed by quantitative real-time polymerase chain reaction (qRT-PCR) (Figure S2). ACA1_153540 showed strong homology to the NAD-dependent deacetylase sirtuin protein-encoding family. Sirtuins are a group of proteins that possess either mono-ADP-ribosyltransferase, or deacetylase activity within the class III histone deacetylase family which couple lysine deacetylation with NAD hydrolysis (Nakagawa and Guarente, 2011). Through this reaction, sirtuins influence a number of cellular processes in mammalian cells including apoptosis, inflammation, transcription and DNA damage. In humans seven types of sirtuins have been identified and fall into four classes (Nakagawa and Guarente, 2011).

In order to examine the evolutionary conservation of the sirtuin protein family in *A. castellanii*, orthologous sequences were identified by BlastP searches against *Homo sapiens, Mus musculus, Caenorhabditis elegans, Saccharomyces cerevisiae* and *D. discoideum*. Seventeen *A. castellanii* proteins were identified and these were aligned using ClustalW, and a corresponding phylogenetic tree was generated using maximum likelihood phylogenetic inference (Figure 5B). The main clades obtained correlated well with the four classes of sirtuin proteins identified in previous studies (Frye, 2000; Greiss and Gartner, 2009). At least one *A. castellanii* protein showed homology to each sirtuin class and the proteins were annotated accordingly. In addition to *sir6f* (ACA1_153540), *A. castellanii* genes *sir4a, sir5a, sir5b, sir6e*, and *sirlikeB* were upregulated from 8 to 24 h post infection (Figure 5C). *sir2a, sir3b*, and *sir6a* were upregulated at 24 h, while *sir7a* and *sirlikeA* were upregulated at 8 and 24 h (Figure 5C). The significant conservation and consistent upregulation of the sirtuin family of genes may be indicative of a critical role in the *A. castellanii* response to *L. pneumophila* infection.

**Figure 6:**
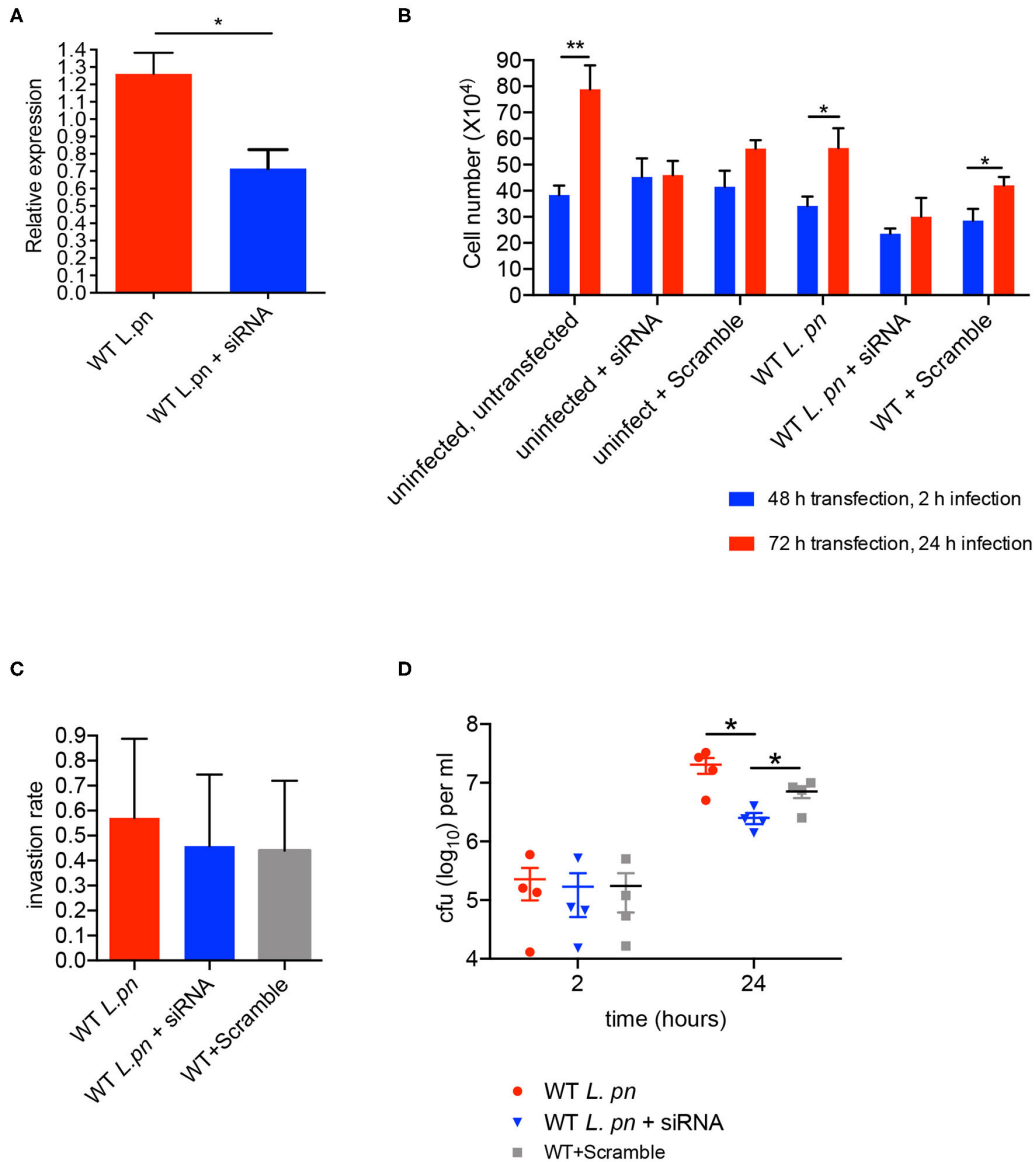
*sir6f* knockdown in *A. castellanii* leads to inhibition of *A. castellanii* proliferation and reduced *L. pneumophila* replication. *A. castellanii* cells were transfected with siRNA targeting *sir6f* or the siRNA scramble control and then infected with wild type *L. pneumophila*. Untransfected *A. castellanii* cells were infected with wild type *L. pneumophila* as a control. **(A)** 24 h post infection sir6f knockdown was confirmed by qRT-PCR. Results were normalized to expression levels of the housekeeping gene, 18s rDNA, and expressed relative to the siRNA scramble control. **(B)** 24 h (2 h infection) and 48 h (24 h infection) post transfection the number of *A. castellanii* cells per well were quantified. **(C)** *L. pneumophila* invasion efficiency of *A. castellanii* following siRNA transfection. Invasion rate was calculated by dividing *L. pneumophila* CFUs 2 h post infection by the inoculum. **(D)** Intracellular replication assay of *L. pneumophila* in *A. castellanii* following siRNA transfection, CFUs were enumerated 2 h and 24 h post infection. All error bars represent the standard error of the mean, where *n* = 4 and *p < 0.05, **p < 0.01.

To determine whether *sir6f* was necessary for the restriction/promotion of *L. pneumophila* replication and/or affected *A. castellanii* viability, we transfected *A. castellanii* with FITC-tagged siRNAs targeting *A. castellanii sir6f* and compared transfected *A. castellanii* to untreated *A. castellanii* and *A. castellanii* treated with a non-targeting scramble siRNA control (optimized for use in human cell lines). siRNA transfection efficiency was optimized by confocal microscopy (Figure S3), and gene silencing of *sir6f* was confirmed by qRT-PCR (Figure 6A). *A. castellanii* cell viability was confirmed using Trypan Blue and showed that silencing of *sir6f* did not result in increased *A. castellanii* cell death (Figure S4). Quantification of *A. castellanii* proliferation at 2 h (48 h siRNA transfection) and 24 h (72 h siRNA transfection) after *L. pneumophila* infection showed that *A. castellanii* proliferation was inhibited when *sir6f* was silenced (Figure 6B). Transfection of uninfected *A. castellanii* with the scramble control also led to the inhibition of *A. castellanii* proliferation, suggesting that the transfection procedure affected *A. castellanii* proliferation. In contrast, tranfection of *L. pneumophila* infected *A. castellanii* with the scramble control did not lead to the inhibition of *A. castellanii* proliferation (Figure 6B). Quantification of *L. pneumophila* colony forming units (CFU) at 2 h and 48 h post infection showed that *L. pneumophila* invasion rate was unaffected by the silencing of *sir6f* , but *L. pneumophila* replication was significantly reduced (Figures 6C,D). This suggested that *sir6f* expression may play a role in *A. castellanii* proliferation during infection and that *L. pneumophila* upregulates *sir6f* to promote bacterial replication.

## Discussion

*A. castellanii* is the natural replicative niche of a wide range of microbial pathogens, including *L. pneumophila* (De Jonckheere, 1991). Outbreaks of Legionnaire's Disease have been attributed to human exposure to *A. castellanii* that harbor replicating *L. pneumophila* from artificial water systems (Best and Abu Kwaik, 2018). Here we have taken a global transcriptional approach to investigate how *A. castellanii* responds to *L. pneumophila* infection. Through RNA-sequencing we found that over the course of the infection a subset of cell trafficking, signaling, metabolism and replication associated genes undergo significant differential expression in a time-dependent manner. These transcriptional responses to infection show similarities with *D. discoideum* and mammalian macrophage responses to *L. pneumophila* infection (Farbrother et al., 2006; Bozzaro and Eichinger, 2011; Hochstrasser and Hilbi, 2017; Kjellin et al., 2019). It is important to note however, that comparisons between different host responses should be taken with caution, as infection protocols vary between studies (i.e., MOI, infection time points and incubation media). Additionally, *L. pneumophila* replicates within *A. castellanii* at multiple temperatures, from approximately 20°C in the natural environment, to up to 40°C in artificial water sources. In this study, infections were performed at 37°C, reflecting the temperature of contaminated man-made water sources. Unfortunately, we were unable to gain sufficient replication of *L. pneumophila* strain 130b at lower temperatures to perform these experiments reliably. Ideally this study would be replicated at a lower temperature reflecting the natural environment for comparison.

Clustering of differentially expressed genes in *L. pneumophila*-infected *A. castellanii* highlighted temporal trends in gene expression and associated biological processes that changed over the course of infection. For the most part, genes that were upregulated or downregulated upon wild type infection remained that way, but the level of expression changed over time. Strikingly, the number of genes differentially expressed from 3 to 8 h increased by 41.5% (5% false discovery rate [FDR] cut-off and adjusted *p* < 0.05). This change has also been observed in *L. pneumophila* infected *D. discoideum* and is likely in response to LCV establishment and the secretion of *L. pneumophila* effector proteins (Kjellin et al., 2019). Importantly, it is understood that *A. castellanii* can exist in different states of ploidy, influenced by environmental factors (Byers, 1979; Maciver, 2016). This polyploid organization of the *A. castellanii* genome may bias expression data if the distribution of ploidy states differs significantly between compared amoeba populations. Previous studies have been unable to conclusively determine the ploidy status of *A. castelanii* trophozoite cultures (Matsunaga et al., 1998; Maciver, 2016). Moreover, it is unclear how bacterial infection impacts the genetic composition of *A. castelanii*. Currently, there is no accepted methodology to correct transcriptional data for potential differences in ploidy. However, future studies investigating individual genes identified as differentially expressed will need to take copy number variation into account.

In mammalian macrophages, the manipulation of small GTPases by *L. pneumophila* is critical to intracellular niche formation and the modulation of host actin cytoskeleton (Machner and Isberg, 2006; Neunuebel and Machner, 2012; Hilbi et al., 2014). In this study, we also identified a number of amoeba genes that show homology to putative small-GTPase encoding genes based on GO ontology and phylogeny. Genes with strong homology to Rho family GTPases showed significant upregulation of gene expression during *L. pneumophila* infection, implicating the regulation of actin dynamics in infection, which is important for phagocytosis (Wennerberg et al., 2005; Swanson, 2008). Actin processing genes included a putative Rho GTPase encoding gene (ACA1_384370) and Wiscott-Aldrich syndrome protein (WASP) encoding gene (ACA1_234760), similar to actin-related genes upregulated in human monocyte-derived macrophages infected by *L. pneumophila*, including WASP factor 1 (WASF1) and Rho GTPases and their effectors (Rac1, RhoA, RhoGAP1, ROCK1, DOCK2) (Price and Abu Kwaik, 2014). To date, several *L. pneumophila* effectors have been identified that target host actin and either promote (VipA) or inhibit (Ceg14, LegK2, and RavK) actin polymerisation (Franco et al., 2012; Guo et al., 2014; Michard et al., 2015; Bugalhao et al., 2016; Liu et al., 2017). Whether the changes in gene expression observed in this study are the consequence of direct bacterial manipulation of host small GTPases or a compensatory upregulation by *A. castellanii* remains to be seen.

During macrophage infection *L. pneumophila* effectors intercept ER-Golgi transport within the first 2 h, recruiting regulators of ER- Golgi transport, such as the small GTPases Rab1 and Arf1 to the LCV (Nagai et al., 2002; Derre and Isberg, 2004; Kagan et al., 2004; Tan and Luo, 2011; Neunuebel et al., 2012). This facilitates the binding of tubular secretory vesicles to the LCV. By 6 h, the LCV membrane resembles rough ER and gradual inactivation and removal of Rab1 from the LCV is observed (Swanson and Isberg, 1995; Kagan and Roy, 2002; Ingmundson et al., 2007; Hubber and Roy, 2010; Neunuebel et al., 2011). During *D. discoideum* infection, the LCV rapidly acquires phosphoinositide (PI) lipids, including the derivative phosphatidyl-inositol (PtdIns) from PtdIns(4)*P*-positive Golgi-derived vesicles. While the initial interaction between the LCV and PtdIns(4)*P*-positive vesicles does not require *L. pneumophila* effectors, the ongoing interaction does (Weber et al., 2014, 2018). Two groups of genes enriched for organelle transport were upregulated throughout *L. pneumophila* infection of *A. castellanii*. The first group, which includes Ras-1-like GTPase encoding gene, ACA1_347020, is predicted to be involved in ER-Golgi transport with peak expression at 24 h. The second, containing hypothetical protein encoding genes putatively involved in transmembrane transport, showed peak expression at 8 and 24 h. Consistently we also observed a progressive upregulation of genes associated with lipid localization over the course of infection, including a putative Ras-related Rap1 encoding gene, ACA1_276770. Rap1 has been shown to localize to the LCV membrane during the infection of *D. discoideum* and mammalian macrophages, and is important for the intracellular replication of *L. pneumophila* (Schmölders et al., 2017). Interestingly, the Ras homologs in *A. castellanii* were upregulated during infection at 8 h and 24 h but not 16 h, perhaps correlating with primary and secondary rounds of LCV biogenesis. The Rab1 homolog, ACA1_175130, and Arf1 related protein encoding genes in *A. castellanii* were also significantly upregulated throughout infection. Future experiments will investigate which small GTPases are important for secretary vesicle recruitment to the LCV during *A. castellanii* infection.

In contrast to cell signaling and transport associated genes, several *A. castellanii* genes involved in metabolic processes were downregulated substantially at 8 h post infection and remained so at 16 and 24 h. Enriched biological processes in these subsets included fatty acid oxidation and steroid biosynthesis. *A. castellanii* genes involved in other metabolic processes such as carbohydrate biosynthesis and nucleoside metabolism showed a gradual reduction in relative expression after 8 h. Genes associated with methane and chorismate metabolic processes were progressively downregulated till 16 h, after which these changes slowed. Consistent with a reduction in energy metabolism, we also observed a downregulation in mitochondrial respiratory chain complex III assembly associated genes from 8 h. The respiratory chain complex III anchors to the inner membrane of the mitochondria and plays a role in the generation of ATP. A reduction in ATP production/respiration associated gene expression has also been observed during *L. pneumophila* infection of *D. discoideum* and coincides with the ability of *L. pneumophila* to reduce mitochondrial respiration and the cellular ATP pool in macrophages by 6 h (Escoll et al., 2017; Kjellin et al., 2019).

In addition to the downregulation of genes associated with *A. castellanii* central carbon metabolism and energy production, we observed the progressive downregulation of genes involved in the replicative cell cycle over the course of the infection, including DNA-dependent DNA replication, mitotic cell cycle, and microtubule cytoskeleton organization. This is consistent with the reduced cell proliferation and cell cycle arrest observed 8 h after *L. pneumophi*la infection (Mengue et al., 2016). Indeed, by 48 h wild type *L. pneumophila* infection of *A. castellanii* resulted in a significant reduction in amoebae numbers compared to infection with Δ*dotA* or in uninfected cells (Mengue et al., 2016). Previous studies in both *A. castellanii* and human macrophages have confirmed that the reduction in host cell numbers is the result of cell cycle arrest and in murine macrophages occurs in a Dot/Icm dependent manner involving the *Legionella* Dot/Icm glucosyltransferase effectors, Lgt1-3 (Mengue et al., 2016; de Jesus-Diaz et al., 2017; Sol et al., 2019). We have now identified a number of genes that may be fundamental to the *A. castellanii* cell cycle.

In this study newly curated GO term allocations were based on homology to annotated genes across kingdoms to account for inter species horizontal gene transfer. Nevertheless, as an understudied organism, a number of the genes remain hypothetical and GO term allocations were fairly broad, based primarily on sequence similarity and domain conservation. Therefore, to identify additional upregulated genes of interest that may have been missed using GO enrichment parameters, we inspected the expression profiles of the Top 20 most upregulated genes from C1. The NAD-dependent deacetylase sirtuin encoding-gene *sir6f* (ACA1_153540) was most upregulated and belongs to a conserved family of sirtuins. In humans, sirtuins are mono-ADP-ribosyltransferase, and/or deacetylases of the class III histone deacetylases which are the only deacetylases that require NAD for their enzymatic function (Imai et al., 2000). Sirtuins are involved in the regulation of autophagy, fat mobilization and tubulin deacetylation (Class I), stress responses and energy metabolism (Class II and Class III), and genome stability, base excision repair of DNA damage and RNA polymerase activation (Class IV) (Nakagawa and Guarente, 2011). Here, 17 sirtuin-like encoding genes were identified, including homologs in each of the four classes of sirtuin. 65% of identified *A. castellanii* sirtuin genes were upregulated upon wild-type *L. pneumophila* infection compared to Δ*dotA*. We found that silencing of *sir6f* inhibited *A. castellanii* cell proliferation during infection but did not affect cell viability. This is consistent with Sir2D knockout strains of *D. discoideum* that show reduced cell proliferation (Lohia et al., 2017). However, *sir6f* silencing in *A. castellanii* did result in reduced *L. pneumophila* replication, suggesting that the upregulation of *sir6f* during *L. pneumophila* infection is advantageous to the pathogen, although the mechanism is unknown. The contribution of sirtuins to *L. pneumophila* replication in human cells is unknown and should be tested in further work.

In conclusion, this study highlighted a number of biological processes in *A. castellanii* that were differentially regulated during *L. pneumophila* infection. A number of genes involved in *A. castellanii* cell cycle, metabolism and membrane function may encode potential biocide targets to reduce *L. pneumophila* environmental replication and persistence. In particular, the sirtuin-encoding gene, *sir6f* , important for both *A. castellanii* proliferation during infection and *L. pneumophila* replication could be targeted within plumbing systems independently of human infection to decontaminate infected water sources.

## Data Availability Statement

The original contributions presented in the study are publicly available. This data can be found here: https://www.ncbi.nlm.nih.gov/geo/ GSE154179, GSM4665492 - GSM4665527.

## Author Contributions

PL, DV, SO, VB-W, JY, and SP performed experiments and data analysis. Initial concept, funding and experimental design were undertaken by CS, EH, and SP. All authors prepared and edited the manuscript.

## Conflict of Interest

The authors declare that the research was conducted in the absence of any commercial or financial relationships that could be construed as a potential conflict of interest.
