# Supplementary material for: Legionella pneumophila Infection Rewires the Acanthamoeba castellanii Transcriptome, Highlighting a Class of Sirtuin Genes
Source: Front Cell Infect Microbiol. 2020 Aug 20;10:428. doi: 10.3389/fcimb.2020.00428 (PMC7468528; doi:10.3389/fcimb.2020.00428)
Supplement: Supplementary file 1 [file Table_1.docx]

*Legionella pneumophila* strains used in this study

| Strain | Characteristics | Source/Reference |
| --- | --- | --- |
| *L. pneumophila* 130b | Serogroup 1, Clinical isolate (USA), ATCC BAA-74 | Declerck *et al*. 2005 |
| *L. pneumophila* 130b Δ*dotA* | *dotA* in-frame deletion mutant of 130b | Merriam *et al*. 1997 |
| *L. pneumophila* 130b (pMip-GFP) | 130b carrying pMip-GFP | This study |
| *L. pneumophila* 130b Δ*dotA* (pMip-GFP) | 130b ΔdotA carrying pMip-GFP | This study |

1. Declerck P, Behets J, Delaedt Y, Margineanu A, Lammertyn E, Ollevier F. 2005. Impact of non-*Legionella* bacteria on the uptake and intracellular replication of *Legionella pneumophila* in *Acanthamoeba castellanii* and *Naegleria lovaniensis*. Microbial ecology 50:536-549.

2. Merriam JJ, Mathur R, Maxfield-Boumil R, Isberg RR. 1997. Analysis of the *Legionella pneumophila* fliI gene: intracellular growth of a defined mutant defective for flagellum biosynthesis. Infect Immun 65:2497-501.
