# Supplementary material for: Legionella pneumophila Infection Rewires the Acanthamoeba castellanii Transcriptome, Highlighting a Class of Sirtuin Genes
Source: Front Cell Infect Microbiol. 2020 Aug 20;10:428. doi: 10.3389/fcimb.2020.00428 (PMC7468528; doi:10.3389/fcimb.2020.00428)
Supplement: Supplementary file 5 [file Data_Sheet_1.docx]

Uninfected Infected

**Supplementary Figure 1**. Representative Flow Cytometry plots showing the proportion of *A. castellanii* cells infected with GFP-expressing *L. pneumophila* (MOI 40) at 24 h. X and Y axis indicate GFP signal and FSC-H (forward scatter height), respectively.


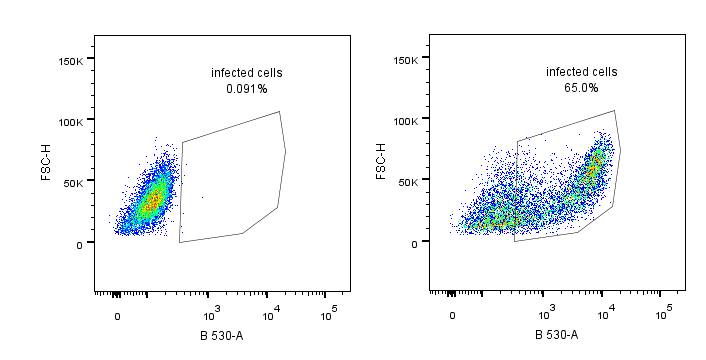


GFP

GFP

FSC-H

FSC-H
