# Supplementary material for: Legionella pneumophila Infection Rewires the Acanthamoeba castellanii Transcriptome, Highlighting a Class of Sirtuin Genes
Source: Front Cell Infect Microbiol. 2020 Aug 20;10:428. doi: 10.3389/fcimb.2020.00428 (PMC7468528; doi:10.3389/fcimb.2020.00428)
Supplement: Supplementary file 6 [file Data_Sheet_2.docx]

**Supplementary Figure 2.** *sir6f* gene expression during *L. pneumophila* infection of *A. castellanii* quantified by qRT-PCR. Wild type *L. pneumophila* infected *A. castellanii* displayed significant upregulation of *A. castellanii* s*ir6f* compared to *L. pneumophila* ∆*dotA* infected *A. castellanii* at 24 h. Results were normalised to expression levels of 18s and expressed relative to uninfected samples. Error bars represent the standard error of the mean, where n = 4 and *= *p*-value < 0.05.
