# Supplementary material for: Legionella pneumophila Infection Rewires the Acanthamoeba castellanii Transcriptome, Highlighting a Class of Sirtuin Genes
Source: Front Cell Infect Microbiol. 2020 Aug 20;10:428. doi: 10.3389/fcimb.2020.00428 (PMC7468528; doi:10.3389/fcimb.2020.00428)
Supplement: Supplementary file 7 [file Data_Sheet_3.DOCX]

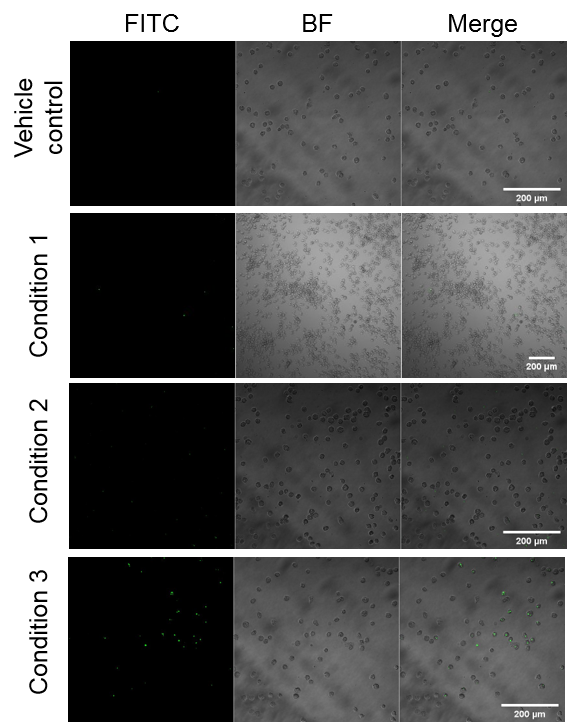


**Supplementary Figure 3.** siRNA transfection of *A. castellanii* visualised by Confocal Microscopy. *A. castellanii* concentration, transfection reagents and siRNA incubation time was optimised before *L. pneumophila* infection. Condition 1: SuperFect-siRNA mixture was transfected to *A. castellanii* (4 × 10^5^ cells per ml) and incubated 72 h before *L. pneumophila* infection. Condition 2: Fugene-siRNA mixture was transfected to *A. castellanii* (1.6 × 10^5^ cells per ml) and incubated 48 h before *L. pneumophila* infection. Condition 3: SuperFect-siRNA mixture was transfected to *A. castellanii* (1.6 × 10^5^ cells per ml) and incubated 48 h before *L. pneumophila* infection. *A. castellanii* transfected with either SuperFect or Fugene was used as vehicle control. Generally, SuperFect generates a higher siRNA transfection efficiency after 48 h incubation (condition 3).
