# Supplementary material for: Legionella pneumophila Infection Rewires the Acanthamoeba castellanii Transcriptome, Highlighting a Class of Sirtuin Genes
Source: Front Cell Infect Microbiol. 2020 Aug 20;10:428. doi: 10.3389/fcimb.2020.00428 (PMC7468528; doi:10.3389/fcimb.2020.00428)
Supplement: Supplementary file 8 [file Data_Sheet_4.DOCX]

**Supplementary Figure 4. Cell viability of *sir6f* silenced *A. castellanii* cells.**

*A. castellanii* viability was measured using trypan blue (Invitrogen™). 100 cells were measured for each sample and the number of live cells presented as a percentage.  *A. castellanii* treated with 4% PFA was used as a positive cell death control. Error bars represent the standard error of the mean, where n=3, and *= *p*-value < 0.05.
